# Comparative effects of a dipeptidyl peptidase-4 inhibitor and of NPH insulin on peripheral nerve conduction of patients with type 2 diabetes

**DOI:** 10.1186/1758-5996-7-S1-A59

**Published:** 2015-11-11

**Authors:** Giordana Maluf da Silva, Carlos Otto Heise, Maria Teresa Hirata, Alberto Andrade de Mello, Katia Camarano Nogueira, Rosa Ferreira dos Santos, Maysa Vieira de Sousa

**Affiliations:** 1 FMUSP, São Paulo, Brazil

## Background

Studies in animals have suggested that the glucagon-like peptide-1 hormone (GLP-1) has neurotrophic properties that were independent of those related to the improvement of glucose control. Dipeptidyl peptidase-4 (DPP-4) inhibitors increase GLP-1 levels and are effective in improving metabolic parameters in patients with type 2 diabetes mellitus (T2D) but little is known about its effects on neurological disorders, including peripheral diabetic neuropathy.

## Objective

To assess the effects of the DPP-4 inhibitor sitagliptin on nerve conduction and their independence on glucose control.

## Materials and methods

Thirty patients with T2D (39-66 yrs.), diabetes duration of 10.9 yrs., inadequately controlled with metformin plus glyburide (HbA1c between 6.9 to 9.1%) were randomized to receive sitagliptin (n=16) or bedtime NPH insulin (n=14) as add-on therapy. HbA1c, fasting blood glucose, body weight and electroneurography were determined before and after 1 year of treatment.

## Results

HbA1c levels decreased both in the sitagliptin (8.01±0.57 x 7.36±1.96, p=0.04) and NPH group (8.11±0.64 x 7.34±0, 68, p <0.001). The weight of patients remained stable. There was no change in sensory and motor nerve conduction parameters in the 2 groups.

## Conclusions

Sitagliptin and bedtime NPH insulin were effective in reducing HbA1c, after 1 year of treatment. The improvement of glucose control, the use of sitagliptin or bedtime NPH insulin did not lead to improvement in peripheral nerve conduction in patients with long-standing type 2 diabetes.

